# Supplementary material for: Outcomes of Kidney Transplantation in Fabry Disease: A Meta-Analysis
Source: Diseases. 2020 Dec 23;9(1):2. doi: 10.3390/diseases9010002 (PMC7838795; doi:10.3390/diseases9010002)
Supplement: Supplementary file 1 [file diseases-09-00002-s001.pdf]

Database: Ovid MEDLINE(R)
Search Strategy:
----
1 exp kidney transplantation/
2 kidney transplant$.mp.
3 exp renal transplantation/
4 renal transplant$.mp.
5 fabry disease.mp.
6 exp fabry disease/
7 lysosomal storage disease.mp.
8 exp lysosomal storage disease/
9 1 or 2 or 3 or 4
10 5 or 6 or 7 or 8
11 9 and 10

****

EMBASE and the Cochrane Database of Systematic Reviews

SEARCH QUERY

('kidney transplantation' OR 'kidney graft' OR 'kidney graft rejection' OR (renal AND transplantation)) AND ('fabry disease' OR 'lysosomal storage disease')
